# System's Crisis Resilience as a Societal Crisis: Knowledge Structure and Gaze of the Finnish Health Care System

**DOI:** 10.1007/s10728-023-00479-3

**Published:** 2024-01-22

**Authors:** Matias Heikkilä, Ossi Heino, Pauli Rautiainen

**Affiliations:** 1 Faculty of Social Sciences, Tampere University, Arvo Ylpön Katu 34 (Kauppi Campus), 33520 Tampere, Finland; 2 Department of Social Sciences/UEF Law School, University of Eastern Finland, Kuopio, Finland

**Keywords:** Counterproductive resilience, Crisis dynamics, Dissensus-seeking Delphi, Health care system, Knowledge structure

## Abstract

The crisis resilience of vital social systems is currently the target of constant development efforts in Finland, as their drifting into crisis would weaken societies’ functional abilities, safety, and security. This is also the case regarding the Finnish health care system. In an attempt to move beyond existing frameworks of crisis imagination, this article takes an unconventional stance by elucidating endogenous crisis dynamics present in the Finnish health care system. Delphi process was conducted for top experts in Finnish health care and crisis management. With a dissensus-seeking orientation, our aim was to fertilize disagreements among panelists to reveal key vulnerabilities in the health system. Despite our efforts to evoke dissensus, the panelists ended up generating a consensus that aims to protect the underlying assumptions of the health system’s knowledge structure. Through inductive analysis of expert discourses, the data was analyzed through our research question “what constitutes a crisis-proof health system and a crisis-prone health system”. What is framed as a strength of the system by our panelists, namely the ability to maintain legitimacy, improve efficiency, and guarantee continuity, can still have questionable implications that are left ungrasped. A system’s theory approach illustrates how such effects can develop and escalate beyond the reach of social interventions, and thus be predisposed to cause objectionable yet concealed social crises. The discussion illustrates how these endogenous crisis dynamics could be seen to materialize in real-life cases.

## Introduction

### Setting the Scene

Health systems are able to tackle a great variety of emergencies and disturbances on an everyday basis, both on a global scale but also on a national level. The global COVID-19 pandemic was tackled both on a global level as well as on national levels by health systems. This has been since studied academically in great detail. The Finnish health system been academically reviewed as having fared well against this global external threat. [21, 41] This ability to maintain essential functions and stability in the middle of a crisis is often referred to as crisis resilience [16, 36]. External threats are thus justifiably a significant area of interest for research and development. But considering how capable many health systems have become in warding off external threats, it could be suggested that other threats and crises should also be imagined and researched.

Most health systems find themselves in a setting where they aim to operate at the height of their abilities with limited resources. In most societies, health in general is of significant societal value. Accordingly, the expectations for health systems can be considerable. To answer this call, health systems aim to optimize, prioritize—often according to their own internal principles and professional knowledge [47]. From a systems theory perspective, this can also lead to other developments besides desired progress.

Approaching systems following sociologist Niklas Luhmann [30], systems possess logic, language and values which are formulated and established through autopoiesis, i.e. through self-determination and differentiation from its environment (pp. 22–25). According to Luhmann, environments are perceived by systems as noise and potential stimuli is detected through the eyes of its own structure (Ibid.). What is known and unknown to a system is conjured through the system’s own logic. Then, if such a “gaze” which orientates a system in its core exists, so must its blind spots. What is then the potential for threats and crisis that lies within the system, around its valued core? Following Alpaslan and Mitroff [1] who state that “every crisis is a deep existential crisis of meaning,” we claim that the core of a system’s endogenous crisis dynamics  can be tied to mechanisms that protect, and also legitimate the permanence of the underlying assumptions. Thus, a system's resilience is not only connected to dealing with external threats, but also connected to the internal, epistemic elements of a system.

To stride beyond threat imagination related to official documents related to the preparedness of the health system, we must first distinguish the limits of the system’s gaze. With the help of top experts from both health care and crisis management serving as informants, we aim to produce discourses that not only reveal the system’s underlying assumptions and how they should be protected, but also open windows to see how power operates around the system’s valuable core (see [10, 11]).

For the boundaries of the system’s gaze to be aptly defined, we organized a Delphi-panel and stimulated our informants with different methods to get them to assess their own gaze in a critical manner. Thus, we decided to fuel dissensus amongst our informants, to reveal the blind spots of their gaze and force them to think outside familiar thought patterns and assumptions. This dissensus-seeking version of the Delphi method gave the informants the opportunity to boldly talk about improbable threat signals, and also to challenge other informants. Lastly, we analyzed the gathered Delphi-data through our inductively formulated research question, namely “what constitutes a crisis-proof health system and a crisis-prone health system”.

Concrete examples of the gaze and its blind spots are from time to time brought to light by the legal system, critical health research, and the media. In the Finnish context, defined by the public health care system, each citizen can invoke their legal right for health services which are guaranteed up to a certain level, dictated by the Finnish legislation. The legal system thus serves to bring some blind spots of the gaze to light for the more general inspection of the public and government officials. One case in point comes from the field of anesthesia dentistry in Finland. Earlier budget cuts in the field’s training led to the dispossession of the severely disabled. The legal system pointed out that this had constitutional implications, but as the root cause of the problem could be tracked to the cutbacks made by the Finnish government, the issue of access to dental care for severely disabled persons was not addressed any further. Within its own parameters, the legal system has the ability to intervene, but in many cases, it can only address consequences–not how the gaze of the health system is directed and motivated. What logic, then, lies behind the gaze? What are the battlefronts where, in the eyes of the top experts in health system and crisis management, the system is strengthened or weakened?

### Gaze Within the Context of the Health System

The intent of the health system is to have a positive impact on a society by promoting, restoring, and maintaining “the health of the population in the most effective manner possible in light of a society’s available resources and competing needs.’^1^ Hence, it shall attempt to “strive for the highest possible health status of the entire population” [12] without doing so at the expense of equity [38, 39]. The goals of the Finnish health care system are stated much in the same way [25], with a focus on the health system providing services according to the law and the temporal goals set by the Finnish ministry of social affairs and health.^2^

That is, the health system should prioritize competing needs and allocate resources in a way that is perceived to be just [13, 15]. Thus, when assessing the operation of the system, a whole range of factors and attributes must be considered to assess whether the system stays on the right track [8, 12]. However, the health system has various formal and informal mechanisms for defining and justifying its approaches. As Knudsen [26] shows, it may, for example, produce self-imposed blindness to potentially harmful information. The information that serves as the basis of the system’s key functions can be filtered by the system itself in many ways.

Since the health system operates in a resource-constrained world [46], it must focus its attention selectively. Moreover, from a systems theory perspective, to maintain its self-renewing capacity, the health system must differentiate itself from its environment [29] by becoming increasingly self-determined in its functions, diagnosis, treatment, and prevention of diseases. An intrinsic consequence is thus that the system strives for autonomy in terms of knowledge and expertise. In turn, it can become increasingly difficult for other social systems to evaluate the health systems’ worldview instead of the mere consequences of its actions. In his groundbreaking book *The Birth of the Clinic: An Archaeology of Medical Perception*, Foucault [9] outlines how “medical gaze’—as a medium of the relationship between knowledge and power—and its development define the functioning and very being of the health system.

Common area of focus in relation to the gaze and the surrounding social systems is embodied in service delivery meeting the demands of “any type of disaster, enabling a coordinated, rapid and effective response and recovery” [2]. In popular discourse, a health system crisis is understood as various disturbances and unwanted deviations in service delivery. However, on the way towards understanding crises beyond the existing crises imagination, inclusive of endogenous threats, a different kind of approach is needed.

### Crisis Undermines System's Underlying Assumptions

Crises are usually thought of as events limited to a clear-cut time and space, most likely caused by external intervention. According to the traditional understanding, as Boin et al. [4] phrase it, “[c]rises explode on the scene but usually disappear into the history books after they have been brought under control”. They are seen as a discrete event which clearly stand out from normal and desired activity, attract attention, and call for responses—they can be detected, identified, and managed. Heino et al. [19] illustrate how difficult it is for the institutionalized preparedness imagination to grasp a crisis that comes with contrary basic characteristics: ambiguous, slowly unfolding, not intentionally caused by anyone. These sorts of crises—if they can even be called crises in the light of a system’s established standards—do not stand out from the background as a viable threat, but rather become part of a system’s function and development that is considered normal, desirable, and legitimate. In other words, a system's functioning is conditioned by its ability to distinguish what should be seen as a figure and what should be considered background. However, as Zerubavel [50] theorizes, “[f]igureness and backgroundness are not inherent qualities […] any component of our phenomenal world can in fact be perceived as *both figure and background.*” But how does the distinction between figure and background relate to a system’s crisis dynamics?

This article discusses the crisis potential of the Finnish health care system and its ability to avoid falling into a crisis. Following Boin et al. [3] who define crises as “episodic breakdowns of familiar symbolic frameworks that legitimate the pre-existing sociopolitical order”, our focus is not crisis definitions related to excess of strain and discomfort within daily operations. The crises that we are most interested in will be directed at the elements of the system that the system perceives as its most vital.

The aim of this study is to shed light on these dynamics in the context of the health system. In order to grasp such crisis dynamics, we must ask: How do the system’s underlying assumptions [1, 32] and familiar symbolic frameworks [3] withstand being questioned or destroyed? To answer this, on the one hand, we aim to elucidate the obvious, foregrounded understanding of how crisis-resilient systems are presumed to function. On the other hand, we exemplify features that go unnoticed, fade into the background, and become normalized and legitimated. To gather information about such a tense setting, we put expert opinions from both the health system and crisis management onto a collision course.

## Materials and Methods

The empirical challenge of such a study is to collect the most fruitful research material, data that would expressively reveal not only the obvious, but also bring into consideration that which is normally hidden. To evoke such empirical data, we employed the Delphi method [28]. This method has been used within health sciences, inter alia, to develop clinical practice guidelines, analyze professional competences, survey quality measures, and enhance public health policy [7, 37]. In contrast to the original method, we sought to fertilize dissensus, i.e., to identify and fuel differences in a panel of experts (on the Argument Delphi Method, see e.g., [27]). This way, we could avoid the harmful side effects of its consensus-seeking relatives, such as the monopolization of discourses, the marginalization of opinions differing from the mainstream, the dominating influence of certain experts, and groupthink [17]. The method allowed us to create the conditions for arguments that underpin the prevailing paradigm of the health system and for counterarguments that criticize them in order to expose vulnerabilities in the system's status quo (cf. [33]). We were also particularly interested in the panelists’ qualitative responses,that is, our objective was to produce fruitful and diverse textual material. Through the tasks assigned to the panelists, we aimed to feed dissensus by contrasting the experts’ arguments with each other and challenging the experts to reflect on their own arguments [20, 31].

**Fig. 1:**
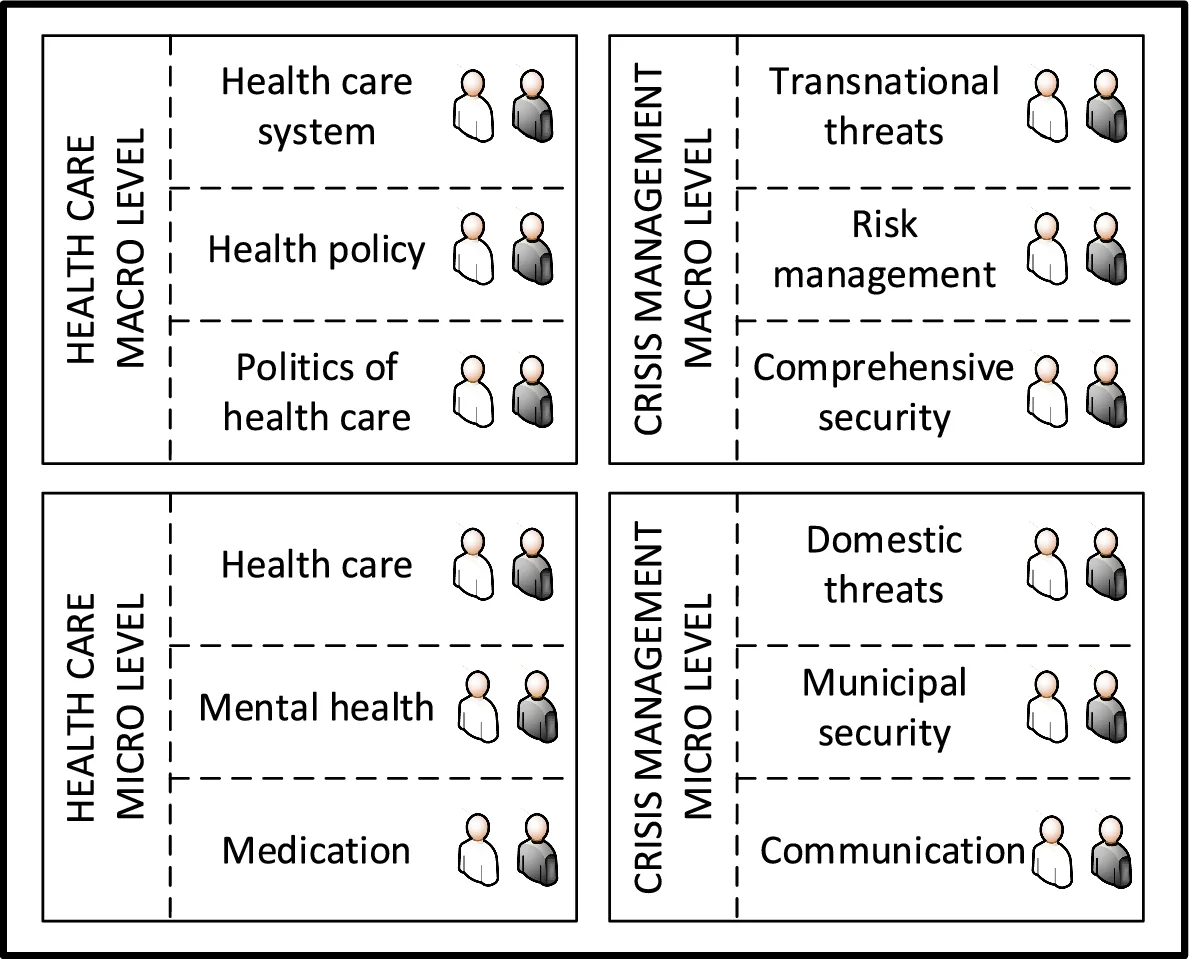
Composition of Delphi panel

A composition of 10–50 panelists is recommended for the dissensus-seeking Delphi method [40]. As our interest focused on qualitative responses, we decided to invite a total of 24 experts, half of whom represented the health system and the other half of whom represented crisis management. Figure 1 shows the expertise matrix used to compile the panel.^3^ We had none of the typical problems of the selection process [18], the invited experts were curiously interested in becoming involved in a study that analyzed the crisis dynamics of the health system. The desire for dissensus was manifested in the way in which we, acting as Delphi moderators, fueled disagreement in argumentation [42].

In the first Delphi round, we presented two fictional scenarios in which we offered hints about how the health system could be heading towards a crisis.^4^ We wanted the questions to encourage the panelists to explicate the actual crisis and how the system should react to it. To sharpen the dissensus, some of the questions were formulated to create clear division amongst panelists that would amount to critique posed against the opposing view. The output from the first round was utilized as input for the second round. The panelists were asked to evaluate different kinds of argumentation and defend the type of argumentation they felt was more legitimate and on sustainable basis, and hence less susceptible to crisis. We created a scenario of a fictional health clinic that combined conventional and alternative medicine. Again, by asking opposing questions, we wanted the panelists to describe their crisis imagination in their answers to what, in fact, constitutes a crisis to the health system In the second round, we pitted some of the differing views of the panelists against each other, again seeking disagreements about what should be allowed within the system and what views and approaches would undermine the system and its crisis resilience.

Following the inductive analysis approach outlined by David Thomas [45] our data led us to detect patterns and discourses that could give us insights into the health system’s crisis dynamics. In our efforts to go beyond the crisis imagination of our panelists, we formulated our research question inductively from the panel data. We analyzed the data through our research question, which we formulated as follows: What constitutes a crisis-proof system and a crisis-prone health system. In other words, we wanted to analyze what dynamics and elements of the system the panelists considered the most vital to a crisis-prone or crisis-proof health care system.

By abstracting discourses that serve as clues for our proper research question, rather than seeking explicit answers to explicit Delphi questions, we began another process of abstraction to then categorize the discourses expressed by the panelists. The final form constituted three discourse categories. We then selected “[a]ppropriate quotations that convey the core theme or essence of a category”, following Thomas’ model [45] . These discourses were used to form a synthesis that would then serve as the answer for our research question and fuel the paper’s discussion (pp. 241–242).

**Fig. 2:**
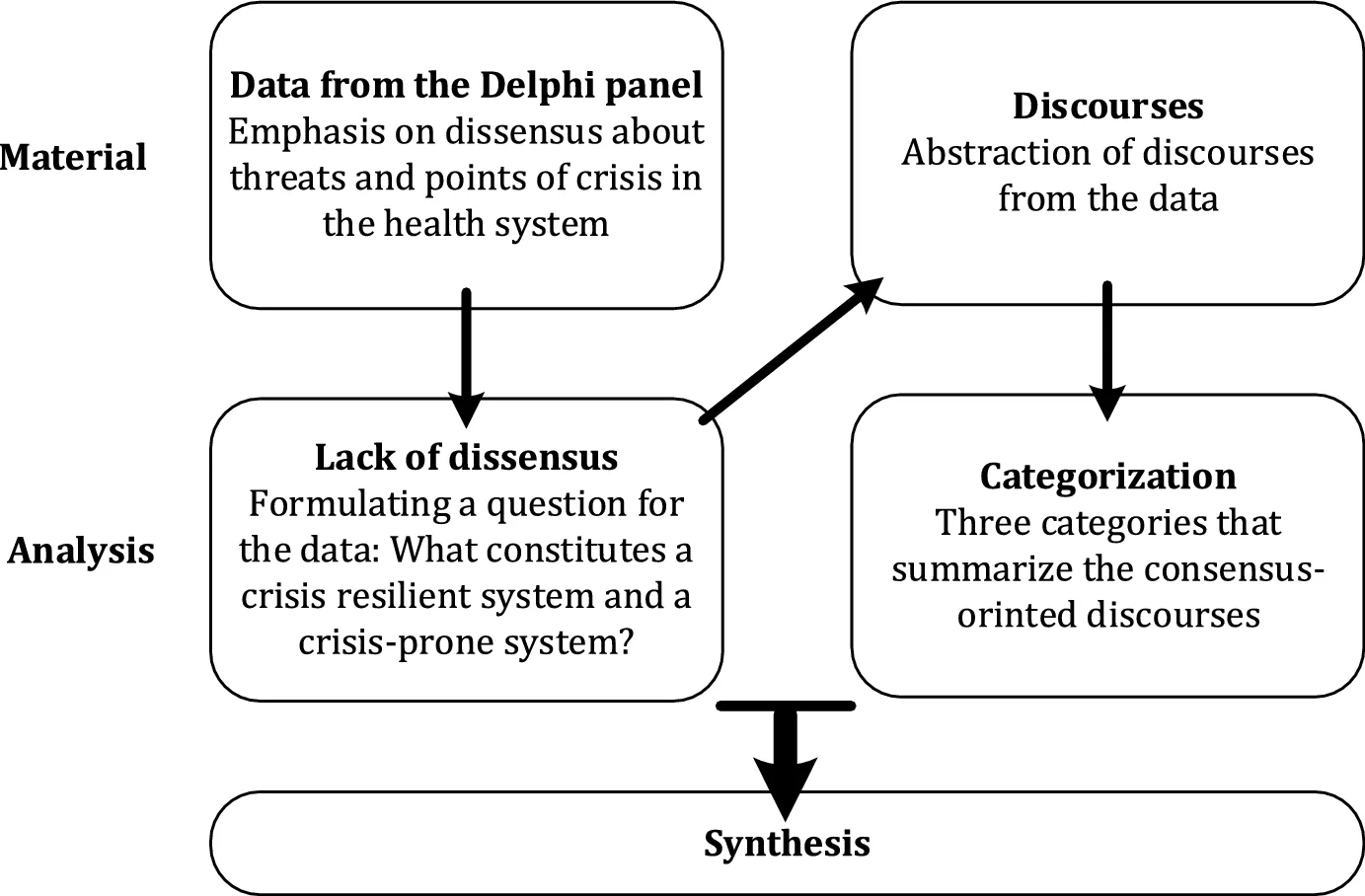
Process of analysis and reasoning

The process of analysis and reasoning is illustrated in Fig. 2.

## Results

### Generating Dissensus-Seeking Discourses on the Health System’s Crisis Dynamics

Regardless of our dissensus-focused approach, it became clear early on that dissensus was not easy to fuel within this context. Whether stemming from the composition of the panel or something else, even our antagonizing tactics of the second round, did not bring about true dissonance. In other words: if the panelists' answers were visualized, the end result would be a curve conforming to a Gaussian distribution, despite the furious desire of the Delphi moderators to pull the graph to its extremes. Fueling a variety of discourses generated by the panelists on the threats, crises, and ground rules of the Finnish health care system, we endeavored to determine what actually constitutes a crisis-prone or crisis-resilient health system.

### Knowledge Structure as the Protected Core of the Health System

One theme proved to be the cornerstone across various schemes and scenarios presented to the panelists: the health system’s knowledge structure. It appears that the panelists' argumentation of each topic ultimately boiled down to a system-oriented view that follows a commonly shared view of the health system. It looks like a crown jewel, from which the power, unity, and continuity of the health system are drawn, and which ought to be guarded vigorously. Anything that threatens the knowledge structure is considered a threat to the existence of the system itself. Therefore, the system's knowledge structure must be self-produced on the basis of its own premises. What kind of underlying assumptions, then, does the system's protected site, its knowledge structure, consist of? The research data revealed three different, yet not exclusionary assumptions:

- Knowledge is pure and plentiful (state-of-the-art knowledge, under the direction of medical science)
- The use of knowledge is disciplined (professionals lean on their education, rely on a higher authority)
- The system is distinct and self-determining in the epistemic sense (defined limits, maintains asymmetry of information to actors outside of the system)

The panelists’ replies illustrated that behavior which threatens these underlying assumptions should not be allowed. In fact, it seemed almost inconceivable to them that something like this would occur within the health system, at least on a broad scale. The panelists’ lines of argument boiled down to describing a health system that held these impressive attributes. This was not only a normative description but also a realistic one, in the view of the panelists.

The pure and plentiful knowledge in the first category included an explication that implied that the health system always relied on state-of-the-art knowledge and was ever evolving under the strict guidance of medical science. Applicable and relevant tests were to be carried out before treatments or medicine were allowed to be used within the system and everything was subjected to the strict rule of medical science. Knowledge as objectively produced; it systematically developed and refined, hence becoming richer and more complete, yet deriving from the autonomy and independence of the system. Thus, the system's knowledge base was not errorless, but identified errors—defined by the system itself—were corrected in the process of knowledge generation.

Medical science, for me, is hard science based on quantifiable and repeatable strict research (nursing science admittedly less so), and if ‘faith’ in that falls apart, it has structural consequences all the way up to the basis of the welfare state and the state as a convention. (Crisis management, micro level)

Diagnoses and treatment decisions must be based on evidence or at least well-documented experience—anything else is quackery and unwarranted. Medical professionals should not interpret the patients’ symptoms themselves on the basis of the position of the stars. (Health care, macro level)

The second category holds statements that call for the enforcement of reliance in the hierarchies of the health system and the medical system, which also go hand in hand. This means that someone higher up on the professional hierarchy deserves their position on the basis of their accumulated health and medical knowledge. These are also the same actors who are allowed to make decisions about which treatments, practices, and medicine are permitted into the system. This also includes such decisions that are not allowed to be formulated in a bottom-up manner. In other words, those higher up in the hierarchy have a sophisticated gaze with which to identify problems appropriately and find the most effective interventions.

The pillars of society, especially health care, must be founded on research-based knowledge. The nature of research-based knowledge is that it progresses through the discovery of better information. Still, health care professionals can’t each look for their own truth. This is not only dangerous; it jeopardizes the credibility of the whole system. (Crisis management, macro level)

One should realize that it’s not only the health of an individual that is at risk but also the functionality of society, as many benefits and institutions are based entirely on medical judgement. For example, mental examination ordered by courts and social benefits based on medical reasons. Also, in a broader sense, the right to life and health of the most vulnerable people would be threatened. (Health care, micro level)

The third category in defining the elements of a system resilient to various types of crises is the system’s ability to self-determine, that is, to set its own distinctive limits and borders within which it may operate. This means maintaining a clear distinction of which actors, services and operative views are prevalent within the system and which should be kept outside of the system. This applies to the leeway with which health professionals must operate in their day-to-day practice. It also applies to the asymmetry of knowledge that needs to be maintained between professionals within and laymen outside the system. In other words, the crisis-resilient system has control over what are considered alternative approaches to health knowledge and practices.

It's impossible to control misinformation by denying it; the hard core of [medical] professionalism is found in the experts' ability to discuss the different dimensions of knowledge, beliefs, and lies. In this way, the professional can offer their expertise to help the patient’s way forward (Health care, micro level)

Even though there is no single version of the truth, cases of abruption or disturbance can’t be dealt with by taking every different opinion into consideration. If there is no official line, a crisis can’t be dealt with in an orderly fashion. The official line can be right or wrong, but establishing one is crucial. It’s vital that people toe the line so that the existing modes of operation remain in operation. (Crisis management, macro level)

The health system must be able to effectively distinguish itself from its environment. For this, it must be able to determine its own epistemic boundaries because otherwise the system would merge with its environment, becoming an ambiguous entity whose autonomy would soon be questioned. In this sense, the epistemic boundaries themselves embody the sophistication of the crisis-resilient system and its ability to identify threats and prepare for them. What is not problematized, however, are the weaknesses associated with clear boundaries.

### Eagerness for Consensus as a Prerequisite for the Pillar of Society

The reason why the three categories appear so clear and steadfast is the pivotal role of the health system as a pillar of society. According to the Finnish constitution, public authorities must ensure adequate social and health services for everyone; this can be considered a fundamental aspect of the Finnish welfare model. Interestingly, though, when the panelists argued the questions we addressed to them, they reflected on the most extreme and purest forms of health care, namely heart bypass surgery or ventilator-assisted treatment in intensive care.^5^ The purest form of health care is what must ultimately be secured, and all other aspects of the health system shall be compared against it.

The loss of society’s trust in [health care] professionals could spread to other fields as well. This could have serious consequences for complying with regulations and the rule of law. (Crisis management, macro level)

The authority of medical science (not so much the authority of the health care system) is such an integral part of the authority of modern institutions—in addition to health care there are ethical codes for work, exercise, and substance use—that the crumbling of this authority would have society-wide reproductions. (Health care, macro level)

The explications in the three categories can therefore be considered a way of legitimizing the gaze. To be able to serve its function as one of the pillars of a society—upon which various legislative decisions, such as sickness indemnity, work disability pension, referral for coercive treatment, etc.—and to be worthy of society's funding, it is abundantly clear that the system’s underlying knowledge structure must enjoy unquestioned confidence throughout society. This not only enables the system to become a consolidated pillar of society, but also brings legitimization to the system’s decisions, such as prioritizing under scarce resources.

This seems to also apply to our themes of consensus and dissensus. There was a clear consensus about the main issues, that is, the threats and the strengths*—*even though a wide range of different approaches to strengths and weaknesses were presented. Even when pitted against an expert’s contrasting argument, the “line was toed”. This is not to say that there was a total lack of different opinions. However, such disagreements tended to focus on the means by which a commonly shared goal could be reached.

### What, then, Threatens the Health System?

Considering all the above, what could be a threat to the health system, and why? In the Delphi process, we provided our panelists with a range of inputs into various potential threats. We draw the following conclusions from their explications. An alternative medicine treatment method might not be a threat in itself. Basically, it is up to each person to decide what kind of treatments or medicines they expose themselves to. However, such forms of treatment should not be included in the scope of the official health system, as it would contaminate its knowledge structure. Doctors or nurses acting out of line might not be a threat by themselves, but they may have already threatened the hierarchical structure, which is based on knowledge. Wearable health technology might not be a threat in itself, but it might pose a threat to the knowledge disbalance between the doctor and the patient. Interestingly, very expensive treatment technologies, which limit the possibilities of access to treatment, were not considered a threat at all. That is, obstacles to access to treatment and the resulting processes of social inequality did not attract attention in the sense of a threat: As there are no mutually conflicting interpretations of the problems, there is no threat.

In summary, the main area of explication was knowledge and its various dimensions. These were perceived as key elements of the current health system as well as the site of numerous ongoing struggles with many battlefronts which pose a threat to the system’s vitality and future. Winning these battles also constituted progress, the system’s desirable way forward, which could be achieved by pushing the enemy lines farther. Recapitulating the Delphi panelists’ views, threats posed against the health system can be subdued by (1) relying more on ever-evolving medical knowledge, (2) knowledge-competence based hierarchies, and (3) remaining vigilant on the borders of the system, while (4) serving as a cornerstone for the greater societal good. We now move on to discuss what these results mean in terms of the system's crisis dynamics.

## Discussion

If the underlying assumptions presented above constitute the hard core of the health system's legitimacy, Boin et al. [3] would agree with our panelists that an attack against them would amount to a crisis in the system. It is thus understandable that actors within the system, as well as actors with a stake in the continuity of the current order of the system would strive to prevent this “deep existential crisis of meaning” by avoiding the questioning and destroying of the underlying assumptions [1, 32]. In other words, the panelists’ views are in line with the views of Mitroff and Alpaslan that a crisis is *a moment of world collapse*, where the whole worldview of the health system is crystallized in its knowledge structure. The knowledge structure is the focal point of meaning, through which the system's operations receive an understandable form and direction, and due to its societal position, contributes to the strengthening of social cohesion.

A particularly interesting result is that adjusting the explication served as a kind of strategy, in which the problem in question was argued against using the chosen knowledge base in a way that its implicit, underlying assumptions were either brought to light or left in the dark. It was a way of maintaining operational capability and a way of progressing in the desired direction. However, there are also clear benefits for non-explication. The ability to bring something to the forefront is vital in the same way as the process of leaving something to the background. It is important nevertheless to note that non-explication is not a sign of negligence but rather of sophistication [50]. Mechanisms that keep certain elements hidden away and highlight others, protect the system’s continuity, intactness, and justification of its knowledge structure [14, 35].

The results indicate that, in order to be crisis resilient, the health system must secure the integrity of its knowledge structure. A gaze that distinguishes the figure from the background must be constituted autonomously and in a self-reproductive manner, conditioned by the system's knowledge structure without dominant measures set from the outside and interventions by other social systems. Since there is an unquestionable consensus on this matter, it is important to turn our attention to the background, something that is thereby not communicated (see [5, 34]).

To draw our attention to the background, let us recall the vignette of anesthesia dentistry mentioned in the introduction. As noted, the shortfall caused by the cutbacks led to the dispossession of the severely disabled. That is, the health system produced a solution to the service delivery problem which can be considered rational and consistent with the performance parameters. As is well known, in a resource-constrained setting [46], trade-offs raise ethical problems, which is why it may be in system’s best interest to hide the transparency of the factors behind decisions [43, 44]. The legal system pointed out that this had constitutional implications related to the European Convention on Human Rights and Finland’s national legislation, as adequate health services must be secured for everyone, also complying with international human rights laws. However, since the root cause of the problem can be tracked to the cutbacks made by the Finnish government, the issue of access to dental care for severely disabled persons was not further addressed.^6^

A similar dynamic can be seen at work in another vignette. Due to expensive technology, fertilization treatments can only be offered to a limited extent. Because the supply mismatches the demand, the health system must prioritize who can receive such treatment. Thus, it was decided that only heterosexual couples could benefit from these treatments. The legal system then expressed that leaving female couples without fertilization treatment is morally repugnant. The health system admitted that the practice has discriminatory effects, but as no suitable approaches to correct the supply–demand imbalance had been identified, the practice was allowed to continue.^7^

In a third vignette the legal system questioned the health system’s decision to use a cheaper experimental drug for the treatment of macular degeneration—a medical condition affecting eyesight. The ordinary drug, Ranibizumab, was approved by the legal system but was also expensive. Thus, it was decided by the health system that treatment of the disease could be done with an off-label drug called Bevacizumab, originally intended to treat certain types of cancer. The off-label drug was used for the treatment of those with a lower socio-economic status, while the more expensive drug, which was already accepted by the legal system, was used in the treatment of the more affluent. The legal system pointed out the risks of using the off-label medicine and reprehended the unequal distribution of these risks among patients. Under the threat of sanctions, prescriptions of this off-label drug were halted. However, the unequal targeting of treatment based on the more expensive drugs continued.^8^

These cases exemplify how the Finnish health system is able to regulate the dynamics between the figure and the background. The resulting consequences of the system’s actions are often assessed by other social systems. However, no external system is able address the system's gaze, which is carefully guarded in a crisis-resilient system. The legal system, as an example of an external system that might attempt to address a system’s internal logic and gaze, did not condemn the gaze that allocated the financial deficit precisely to anaesthesia dentistry three decades ago but pointed out the constitutional problem it caused. The legal system did not condemn the heteronormativity of the gaze with which fertility treatments were prioritized but pointed out the resulting unequal effects of the practice. The legal system did not condemn the discriminating gaze but pointed out how the risks of off-label drugs are allocated unequally. The gaze, which remains beyond the reach of external interventions, can fade the highlighted figure back into the background, thus continuing its operations without an outbreak of a crisis.

Our analysis of the discourses produced by the Delphi panelists indicates that protecting the gaze is itself a prerequisite for the health system’s continuity and crisis resilience. This explains why the underlying root causes of our vignettes remain ungrasped. Only the consequences can become politicized as problems and yet, the gaze can still reframe them and fade them back into the background.

When we view the development of the health system from a broader perspective, we can see its capacity for self-reflection. It is no secret that a variety of human suffering and repulsive treatments have occurred through the history of medicine and that at the same time we are reminded that much of it has been a prerequisite for development. This raises the question of whether the health system is capable of deeply, ethically reflecting on its gaze—but usually only retrospectively, after decades [49]. That is, if there is something unethical in the health system, this can usually only come to light retrospectively.

Alternatively, this implies that there is knowledge in the system that is ‘too sensitive, dangerous or taboo to produce’ at the present moment, as it could harm the prevailing perception of the system within a society [23, 24]. DeNicola [6] would describe it as “the construction of a barrier at the boundary of ignorance”. In line with Kempner et al. [22], such information can be seen as revolutionary, because its politicization would undermine the health systems' normative structure—and we would drift towards a social crisis.

This is supported by our findings from the Delphi process. Despite all our provocative attempts, none of the panelists brought up anything unethical or evil in the current system; instead, the system's gaze was perceived as unquestionably ethical and good. Luhmann [30] would conclude here that “a system can see only what it can see” and it also cannot understand what it cannot see (pp. 22–23).

Consequently, the current unquestionability is linked to the system’s ability to stay resilient to crises, which in turn has highly desirable social consequences. In line with Zerubavel [50], the crisis-resilient system manages to define “what we ought to actively *dis*attend”. Ignoring something, in other words, is often a result of more than simply failing to notice it. This, then, is the question our analysis raises for further research and social dialogue: Should deeper ethical self-reflection be demanded of the health system at each time point? If the internal assessment process fails, who should sound the alarm in the case of a social crisis?

## Footnotes

1 https://www.encyclopedia.com/science/encyclopedias-almanacs-transcripts-and-maps/healthcare-systems

2 https://julkaisut.valtioneuvosto.fi/bitstream/handle/10024/164463/STM_2022_18J.pdf?sequence=1&isAllowed=y (abstract included in English).

3 Our panelists hold key positions in state-level governance, health organizations (both public and private), administrative bodies as well as universities. The panel was operated online due to the limitations of the on-going pandemic, through a e-Delphi platform, specifically designed for the Delphi method.

4 The erosion of people’s trust in the health system (increasing vaccine hesitancy, indifferent health behavior, popularity of alternative medicine, etc.); the health system as a subject of information warfare; health care professionals’ skepticism about medicine and expensive technology increases social inequalities and vulnerabilities of wearable health technology (collecting patient information, hacking of devices; treating mental health problems with AI applications).

5 Just as Foucault [9] describes, the medical clinic is at its purest when the patient entering its remit also enters its stage of power, as a helpless object of medical gaze and professional authority.

6 See < Urgent dental care of a severely disabled patient was neglected (oikeusasiamies.fi) > [English] and < https://www.oikeusasiamies.fi/r/fi/ratkaisut/-/eoar/4915/2013 > [Finnish].

7 See < YVTltk-tapausseloste-2019-hedelmoityshoito-syrjinta.pdf > [Finnish].

8 See < Voiko eduskunnan oikeusasiamies määrätä lääkkeitä? (duodecimlehti.fi) > [Finnish].
